# Culturable bacteria diversity in stem liquid and resina from *Populus euphratica* and screening of plant growth-promoting bacteria

**DOI:** 10.1186/s12866-022-02731-7

**Published:** 2022-12-29

**Authors:** Nusratgul Anwar, Yuhang Jiang, Wenbo Ma, Yuhao Yao, Jue Li, Gulibahaer Ababaikeli, Guoqiang Li, Ting Ma

**Affiliations:** 1 Key Laboratory of Molecular Microbiology and Technology, Ministry of Education, College of Life Sciences, Nankai University, Tianjin, 300071 China; 2 College of Life Sciences, Xinjiang Normal University, Urumqi, 830054 China

**Keywords:** Culturable bacteria, Plant-promoting Bacteria, Populus euphratica

## Abstract

**Background:**

*Populus euphratica* Olivier is a kind of tree capable of growing in extremely arid desert and semi-desert environments. In this study, a culture-dependent method was used to analyze the bacterial diversity of stem liquid of *P. euphratica* and resina of *P. euphratica*, and to further evaluate plant growth promoting (PGP) activity.

**Results:**

A total of 434 bacteria were isolated from stem fluid and resina of P. euphratica in Ebinur Lake Wetland Nature Reserve and Mulei Primitive forest. The results of taxonomic composition analysis shows that Gammaproteobacteria, Firmicutes, and Actinobacteria_c are the three dominant groups in all the communities, and the representative genera are *Bacillus*, *Nesterenkonia* and *Halomonas*. The diversity analysis shows that the culturable bacterial community diversity of *P. euphratica* in Ebinur Lake Wetland Nature Reserve is higher than that in Mulei Primitive forest, and the bacterial community diversity of *P. euphratica* stem fluid is higher than that of resina. According to PGP activity evaluation, 158 functional bacteria with plant growth promoting potential were screened. Among them, 61 strains havephosphorus solubilizing abilities, 80 strains have potassium solubilizing abilities, 32 strains have nitrogen fixation abilities, and 151 strains have iron ammonia salt utilization abilities. The germination rate, plant height, and dry weight of the maize seedlings treated with strains BB33-1, TC10 and RC6 are significantly higher than those of the control group.

**Conclusion:**

In this study, a large number of culturable bacteria were isolated from *P. euphratica*, which provides new functional bacteria sources for promoting plant growth.

**Supplementary Information:**

The online version contains supplementary material available at 10.1186/s12866-022-02731-7.

## Introduction

*Populus euphratica* Olivier is a kind of tree with high resistance to high temperature and salinity, which is the only arbor that can grow in the saline-alkali arid desert in northwest China [1, 2]. The stem liquid of *P. euphratica* is one of the main implements to cope with the arid and salinized environment. It absorbs water with its powerful roots, and transports it to the trunk, branches and other places for the stem to cope with drought. *P. euphratica* resina is another way to cope with high salinity. When salt accumulates too much in *P. euphratica*, it secretes yellowish crystals through cracks in the trunk and other places, which is called resina. However, as the habitat is being destroyed, the original *P. euphratica* forests tend to decline, and *P. euphratica* resources are endangered and diminishing. Therefore, it is very important to study the active ingredients that promote the adaptation and survival of *P. euphratica* in a specific environment.

It is generally believed that endophytic bacteria refer to the bacteria that can grow in plants but do not harm them [3]. In addition to colonizing the same niche of phytopathogens, endophytes synthesize secondary metabolites with antimicrobial properties, and induce the systemic resistance and growth of the host plant [4–6]. Endophytic bacteria have great potential for application in different industries such as medicine, biotechnology, and agriculture. They directly or indirectly help the plant grow by providing nutrients such as iron, phosphorus and nitrogen [7, 8], as well as by synthesizing hormones such as indole-3- acetic acid (IAA) and ethylene [9–11]. Due to their myriad functions, endophytes are considered viable alternatives to replace or reduce the use of fertilizers and pesticides [12]. Most endophytic bacteria can coexist and coevolve with host plants for a long time. Studies have shown that many endophytic bacteria can fix nitrogen in the atmosphere [13–15], dissolve and absorb iron from soil [16], and dissolve precipitated phosphate through acidification, chelation, ion exchange, and generation of organic acids [17, 18], which can be utilized by their host plants. Therefore, the ability of bacteria in phosphorus solubilization, potassium solubilization and nitrogen fixation is the main index to evaluate the growth promoting potential of bacteria. *P. euphratica* living in a specific environment contains unique microbial resources, and these microorganisms will affect the growth and development of *P. euphratica*.

In order to study the diversity and biological characteristics of plant endophytic bacteria which are used to promote plant growth and acquire new products and to realize the collection and storage of microbial resources, it is necessary to obtain as many pure cultured bacteria as possible. So far, no reports have studied the bacterial communities in the stem liquid or resina of *P. euphratica*. Despite the availability of more robust tools to unravel the bacterial community such as the metagenomics technique, the use of cultivable microorganisms is still valid because it aids in analyzing the community’s functional trait profile and establishing a microbial bank for bioprospecting processes and products. We hope to have a more comprehensive understanding of the composition and distribution of endophytic bacteria in *P. euphratica*. By using pure culture, we can isolate as many strains as possible. Then we obtain and preserve these valuable bacterial resources for the subsequent researches, and the discovery and utilization of new species.

In this study, we studied culturable bacterial diversity of stem liquid and resina from *P. euphratica* in Ebinur Lake Wetland Nature Reserve and Mulei Primeval Forest. The medium composition and its effect on the isolation of bacteria were analyzed. We also studied the function of the bacterial community of *P. euphratica* and established a microbial group with potential application in agriculture.

## Materials and methods

### Collection and treatment of plant samples

**Fig. 1:**
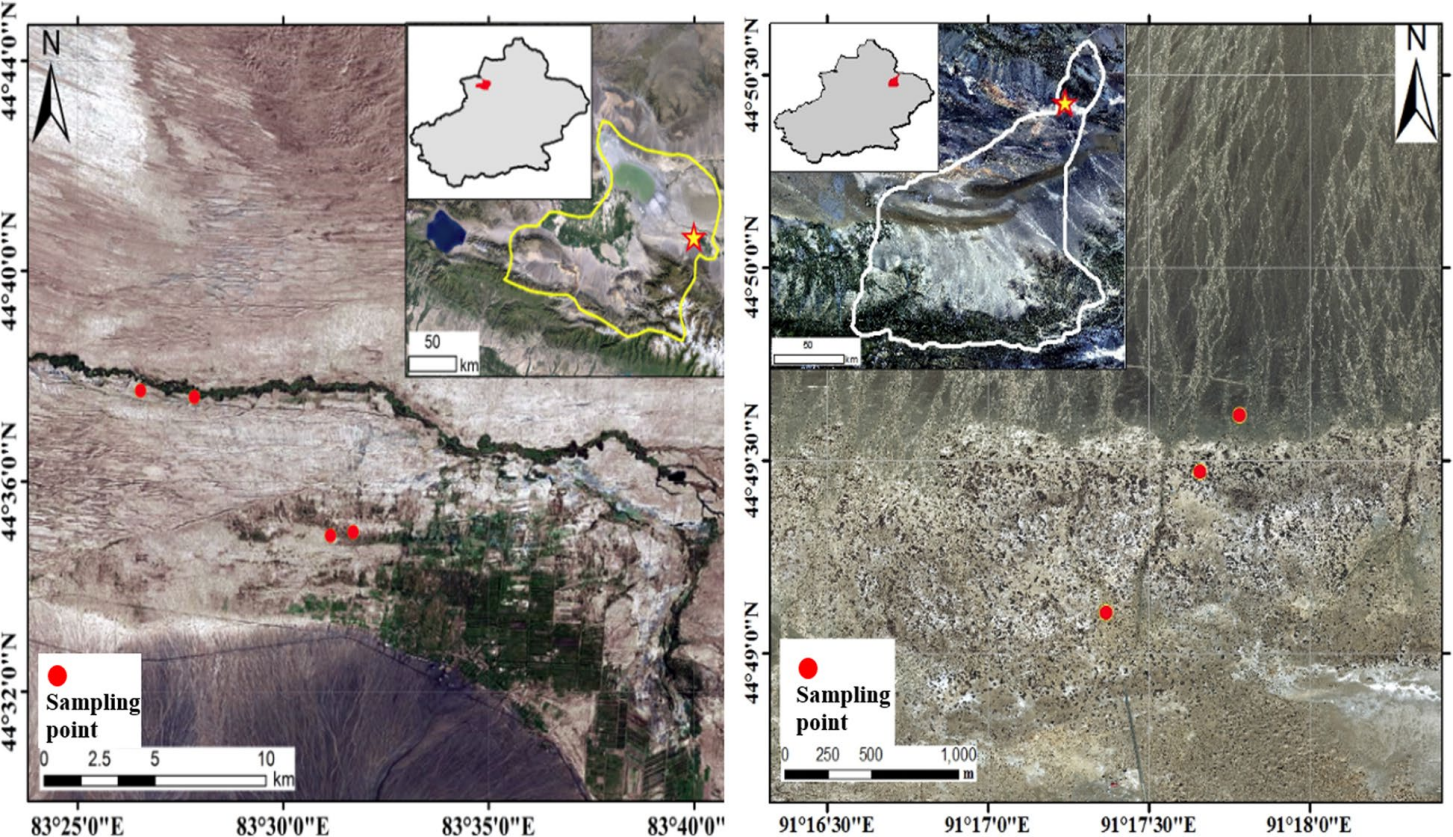
Schematic diagram of sampling sites **A**: 4 sampling sites in Ebinur Lake; **B**: 3 sampling sites in Mulei

On June 18th, 2015, three sites were selected from the *P. euphratica* forest in Yanchi National Public Welfare Forest Area in Mulei County, and three *P. euphratica* trees were selected from each sampling site, as shown in Fig. 1 (N:44°49′6″ E:91°17′22″; N:44°49′28″ E:91°17′39″; N:44°48′37″ E:91°17′23″). On June 25th, 2015, in the east of Ebinur Lake National Wetland Nature Reserve near Toto Township, Jinghe County, four sites of the *P. euphratica* forest in the Aqiksu River basin were selected, and three *P. euphratica* trees were selected from each sampling site, as shown in Fig. 1 (N:44°34′58″ E:83°31′9.1″; N:44°37′36″ E:83°27′50″; N:44°37′43″ E:83°26′31″; N:44°37′53″ E:83°25′46″).

After using 70% alcohol to disinfect the drill and the bark of three healthy *P. euphratica* selected from each sample site, we use the drill to drill holes on the disinfected bark. When the liquid in the *P. euphratica* stem is ejected from the orifice, collect the liquid in a sterilized container and store it at a constant temperature of 4℃. Then use the specially prepared *P. euphratica* wooden stake to fill the drill holes on the tree pole tightly to prevent *P. euphratica* from dying. At the same time, take away a piece of resina on the wound scar of the same *P. euphratica* and keep it in a sterile fresh-keeping bag.

### Isolation of cultivable bacterial strains

According to the available data, only a small number of bacteria are culturable [19]. Moreover, media have a great influence on the number and types of isolated endophytic bacteria [20]. Therefore, to isolate as many bacteria as possible from the stem fluid and resina, according to the characteristics of endophytic bacteria in plants, five media with different chemical compositions and suitable for different bacteria are selected in this study, namely: MA, TSB, R2A, PEA and BPA. (Table S1).

Mix the *P. euphratica* stem liquid collected at each sampling site in equal amounts to prepare compound liquid. Mix 1 ml compound liquid with 9 ml sterile water for 2 h and circulate several times to prepare the stem liquid with different dilutions of 10^–1^,10^–2^,10^–3^,10^–4^,10^–5^ and 10^–6^, and spread on solid PEA, BPA, TSB, MA, R2A medium and incubated at 35–37 ℃ for up to 10 days.

The resina was covered by a thin layer of white salt and located near a wound, where briny water leaked out. Grind the resina into powder, Weigh 1 g from each collected resina, add 10 mL sterile saline and shake for 5 h. According to the conventional dilution-plating method, serially diluted (tenfold dilutions each) samples were made and spread on solid PEA, BPA, TSB, MA and R2A medium and incubated at 35–37 °C for up to 10 days. After the bacteria grow out with obvious colony shape, pick every single colony with unique morphological characteristics to a new same culture media and stored it with glycerol at -80℃.

### Bacterial molecular identification and diversity analysis

Genomic DNA was extracted and purified by using a Wizard Genomic DNA purification kit (Promega). PCR was performed by using universal primer pair 27F (5’-GAGAGTTTGATCMTGGCTCAG-3’) and 1492R (5’-TACGGYTACCTTGTTACGAC-3’) to amplify the 16S rRNA gene. The purified PCR products were cloned into the vector pMD19-T (TaKaRa) and then sequenced. The 16S rRNA gene sequence was used for pairwise sequence alignment performed by the BLASTN program (http://www.ncbi.nlm.nih.gov) and the EzTaxon-e server (http://eztaxon-e.ezbiocloud.net) [21]. Multiple sequences alignment was performed with the CLUSTAL_W 1.8 program of the MEGA 6 package. The evolutionary distances were calculated using the Kimura two-parameter model [22]. Phylogenetic trees were reconstructed from 1000 replicates each for bootstrap analysis with the neighbor-joining methods by using the MEGA6 version [].

Berger-parker's dominance index formula was used to calculate the dominance index of each media [23]. The formula of Berger-Parker's dominance index is: D = N/NT (N: the bacterial species screened in each media; NT: the total isolated bacterial species).

### Screening of functional bacteria with plant growth promoting potential

TO detect the bacteria's abilities of phosphorus dissolving, potassium dissolving, nitrogen fixation, and ferric ammonia salts utilizing, the isolated bacteria were inoculated on inorganic phosphorus medium, silicate bacteria medium, Ashby nitrogen fixation [24] and iron bacteria medium. And they were cultured at 35℃ for 10 days, and significantly, Ashby nitrogen fixation media and iron bacteria media should be put in the dark and inoculated 3 times especially. The phosphorus solubilizing and potassium dissolving abilities were determined by the ratio of a transparent circle to colony diameter. The bacteria that can still grow on Ashby nitrogen-fixation media for the third time are considered as having the nitrogen-fixing ability. The bacteria that can still grow on iron bacteria media for the third time and appear yellow are considered as having the ability to utilize iron ammonium salts.

Inorganic phosphorus media: Glucose 10 g, Ca_3_(PO_4_)_2_ 5.0 g, (NH_4_)_2_SO_4_ 0.5 g, NaCl 0.3 g, KCl 0.3 g, MgSO_4_ 7H_2_O 0.3 g, MnSO_4_ 0.03 g, agar 15 g, FeSO_4_ 7H_2_O 0.003 g, Yeast extract 0.5 g,1000 mL H_2_O, pH7.0–7.5. Silicate bacterial media: sucrose 5 g, MgSO_4_ 0.5 g, CaCO_3_ 2.0 g, Na_2_HPO_4_ 0.5 g, FeCl_3_ 0.005 g, glass powder 1 g, agar 15 g, H_2_O 1000 mL, pH 7.0. Ashby nitrogen fixation media: K_2_HPO_4_ 0.2 g, MgSO_4_ 0.2 g, NaCl 0.2 g, CaCO_3_ 2.0 g, mannitol 10.0 g, CaSO_4_ 0.1 g, agar 15 g, H_2_O 1000 mL. Iron bacterial media: ammonium ferric citrate 10.0 g, (NH_4_)Fe(SO_4_)_2_ 0.5 g, MgSO_4_ 0.5 g, CaCl_2_ 0.2 g,K_2_HPO_4_ 0.5 g, NaNO_3_ 1.0 g, agar 15 g, distilled water 1000 ml, pH 6.9.

### Effects of Populus euphratica culturable bacteria on germination and seedling growth of maize

Bacteria with the above four functions were selected and inoculated in 50 mL MA culture media. After shaking the culture for 24 h, dilute the bacteria solution and adjust the OD_600_ value to 0.5 with an ultraviolet spectrophotometer, and store the dilution for the next use.

Select healthy corn seeds that are ripe, full, and uniform in size at random. Then disinfect them: wash with distilled water, treat with 0.01% Tween20 for 1 min, treat with sodium hypochlorite with 4.5%-5% significant chlorin for 3 min, treat with sterile 2.5% sodium thiosulfate for 10 min, treat with 75% ethanol for 7 min, wash with sterile water for 6 times and treat with sterile 5%/10% sodium bicarbonate for 5 min. After surface disinfection, the seeds were soaked in distilled water for 10 min in a constant temperature water bath at 55℃ and then soaked in the diluted fermentation broth of the strain to be tested for 12 h. Two layers of filter paper and three layers of gauze were placed in the sterilized culture dish, and then 15 infected seeds were placed in the culture dish after being soaked with 10 ml fermentation broth of the filtered bacteria. Sterile distilled water and sterile MA media were used as control groups (CK1 and CK2), and each sample was divided into 3 groups. Germination was accelerated at 28℃ under light (2200Lx, 16 h light /16 h darkness) for 3–5 days, and the breakthrough of radicle through seed coat was used as a sign of seed germination. The germination status of seeds was observed every day and the germination number, bud length, and root length were recorded. Select corn buds with consistent growth and good germination for later use.

The soil used in the experiment was sterilized for 30 min and three times consecutively. Fill each pot with 400 g of sterilized soil. The corn buds infected by bacteria were transplanted into pots, and 8 corn seed buds were transplanted into each pot. Sterile water was applied to the soil, and constant temperature culture was conducted at 8℃ under light (2200Lx, 16 h light /16 h darkness). After emergence (about 3–4 days of cultivation), 5 ml sterile fermentation liquid was added to the rhizosphere of each seedling once every three days. Sterile distilled water and sterile MA media were set as the control group. No fertilizer was applied to all treatments, and sterile water was sprayed in a small amount every day to maintain soil water content. The plant height and dry weight of maize were measured after 20 days’ culture.

### Physiological and biochemical characteristics of growth-promoting bacteria

To study the phenotypic characteristics, growth range, and optimum condition of growth-promoting bacteria, the physiological and biochemical characteristics of common enzyme activities were tested according to *the Handbook of* *Common Bacterial System Identification.*

### Genomic analysis of growth-promoting bacteria

After passing the test, the DNA was sent to Shanghai Bioengineering Co., Ltd. for genome-wide sequencing. After obtaining the original data, the following analysis was conducted: the quality of the sequenced raw data was evaluated by FastQC. Trimmomatic was used to cut the Illumina sequencing data by mass, and relatively accurate and effective data were obtained [25]. SPAdes were used to splice second-generation sequencing data [26]. GapFiller was used to fill gaps in contig [27] PrInSeS-g was used for sequence correction to correct editing errors and insertion missing of small fragments in the previous stitching process. Use Prokka [28] to predict gene elements: genes, tRNA, rRNA, etc. RepeatMasker was used to identify the repeated sequences on the genome [29]. Then use CRT to perform CRISPR predictive analysis. The obtained gene sequences were compared with CDD, KOG, COG, NR, NT, PFAM, Swissprot, TrEMBL, and other databases by NCBI Blast + to obtain the functional annotation information [30]. GO functional annotation information was obtained based on gene annotation results with Swissprot and TrEMBL. Gene KEGG annotation information was obtained by KAAS.

## Results

### Analysis of medium composition and its effect on isolation of bacteria

In this study, a total of 434 bacterial isolates have been obtained, including 258 strains of endophytic bacteria and 176 strains of resina bacteria. Among them, 69 strains were isolated from TSB media, 144 strains from MA media, 52 strains from R2A media, 71 strains from PEA media, and 98 strains from BPA media. The five selected media have different chemical compositions and contents, which are suitable for the growth of different bacteria. Tryptone, NaCl and yeast extract are the main components of most media. MA medium contains trace element mother liquor, R2A medium contains chemical components not found in other media, and BPA medium contains no salt.

The MA medium used in this study was diluted 3 times, and the final amount of NaCl was 20 g; The TSB medium was diluted 2 times, and NaCl was used. The final amount was 30 g; The first-use PEA medium was supplemented with the extract of *P. euphratica* stems (Table S1 is the similarities and differences of various medium components).

**Fig. 2:**
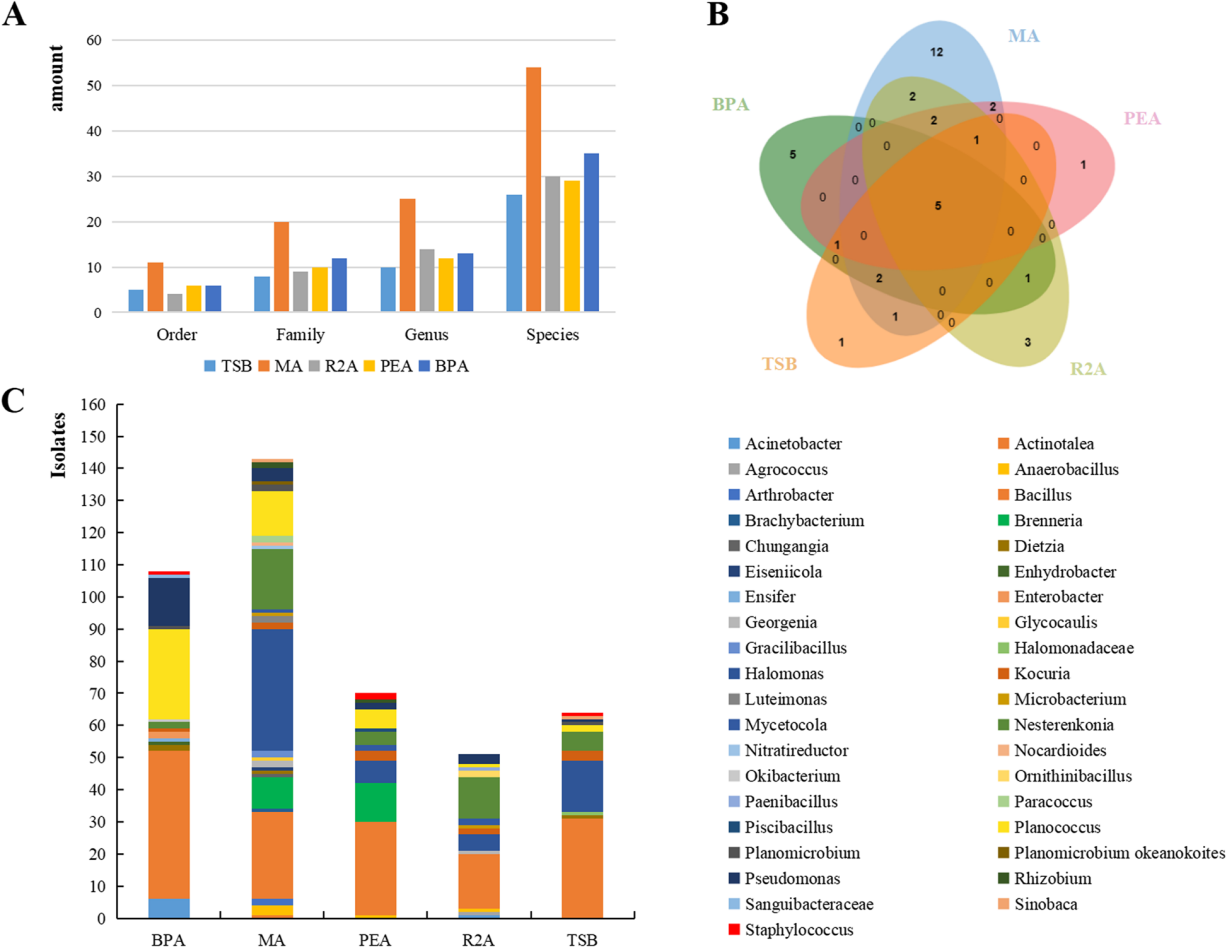
Bacterial diversity isolated from different media. **A**: the amount of order, family, genus, species isolated from five media; **B**: Venn diagram of culturable bacterial genera isolated from five media; C: the number of culturable bacteria isolated from different media at genus level

The strains isolated from the MA medium belong to the following genera: *Actinotalea*, *Bacillus*, *Brachybacterium*, *Brenneria*, *Chungangia*, *Dietzia*, *Chungangia*, *Dietzia*, *Eiseniicola*, *Georgenia*, *Glycocaulis*, *Gracilibacillus*, *Halomonas*, *Kocuria*, *Luteimonas*, Microbacteriu, *Mycetocola*, *Nesterenkonia*, *Nocardioides*, *Paracoccus*, *Pseudomonas*, *Rhizobium*, *Sinobaca*, *Planococcus*; The strains isolated from R2A medium belong to the following genera: *Acinetobacter*, *Agrococcus*, *Anaerobacillus*, *Bacillus*, *Georgenia*, *Halomonas*, *Kocuria*, *Microbacterium*, *Mycetocola*, *Nesterenkonia*, *Ornithinibacillus*, *Paenibacillus*, *Planococcus*, *Pseudomonas*. Strains isolated from TSB belong to the following genera: *Bacillus, Dietzia, Halomonadaceae, Halomonas, Kocuria, Nesterenkonia, Planococcus, Pseudomonas, Sinobaca, Staphylococcus*; The strains isolated from BPA medium belong to the following genera: *Acinetobacter, Bacillus, Dietzia, Enhydrobacter, Ensifer, Enterobacter, Kocuria, Nesterenkonia, Okibacterium, Planococcus, Pseudomonas, Sanguibacteraceae, Staphylococcus*; The following genera were isolated from PEA medium: *Anaerobacillus, Bacillus, Brenneria, Halomonas, Kocuria, Mycetocola, Nesterenkonia, Piscibacillus, Planococcus, Pseudomonas, Rhizobium, Staphylococcus* (Fig. 2)*.*

Isolates belong to 25 genera and 54 species. However, the isolation effect data of the used medium shows that the R2A has the highest dominance index of 0.576, by using it 30 species were obtained from 52 isolates. The followed dominance index is PEA supplemented with *P. euphratica* stem extract with a value of 0.408. The dominance index of TSB was close to that of MA (0.376). The dominance index of BPA was 0.357 (Table 1).

**Table 1:** The composition and dominance index of the five-media used

medium	PH	NaCl	Order	Family	Genus	Species	Strain	sp.nov	Dominant index
TSB	8.0–8.5	30	5	8	10	26	69	23	0.376
MA	6.5–7.0	20	11	20	25	54	144	85	0.375
R2A	7.5–8.0	10	4	9	14	30	52	19	0.576
PEA	7.0–7.5	5	6	10	12	29	71	23	0.408
BPA	7.0–7.5	0	6	12	13	35	98	30	0.357

### The bacterial community in P. euphratica

**Fig. 3:**
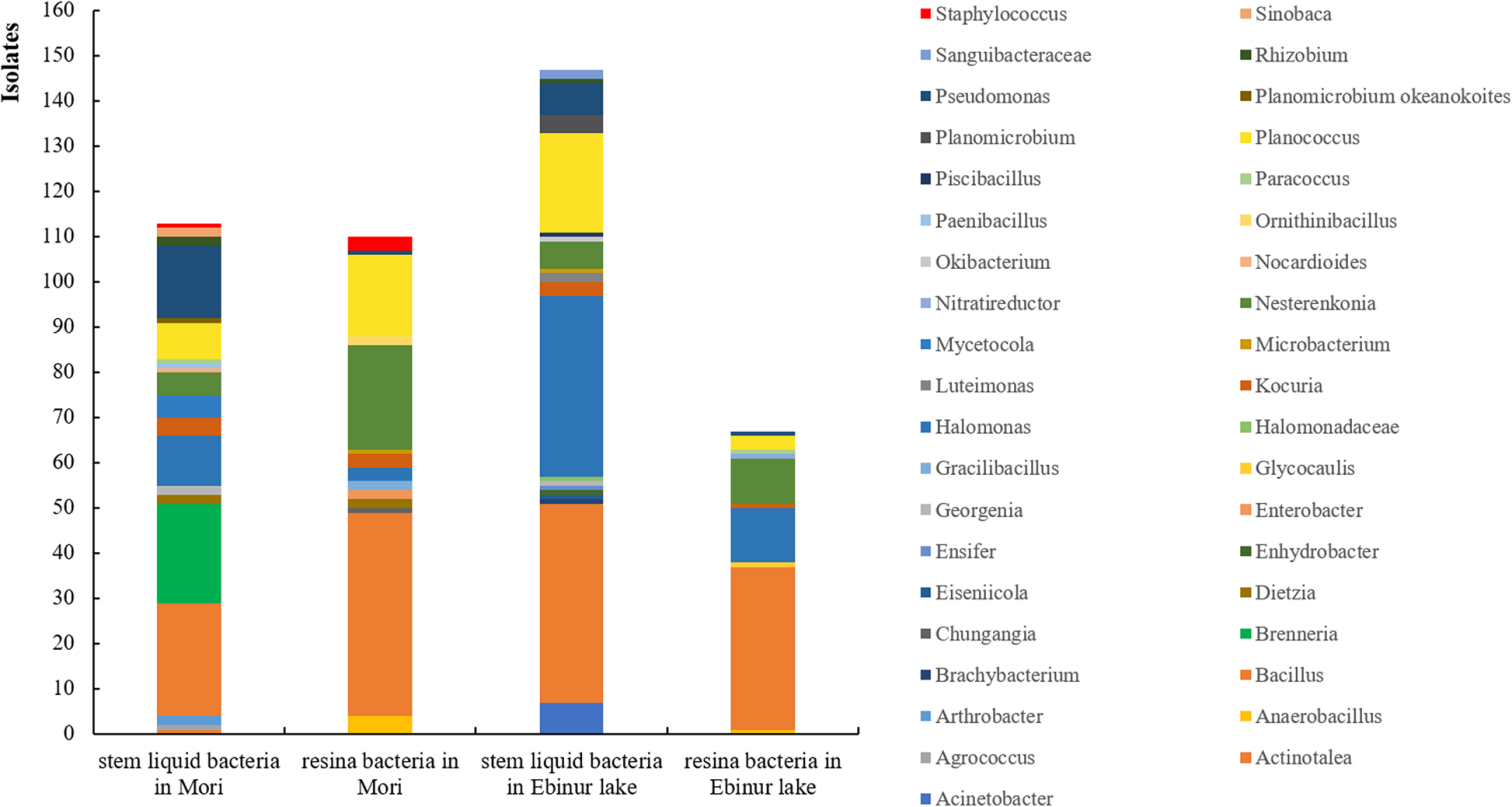
The number of culturable bacteria isolated from different samples at the genus levels

Phylogenetic analysis of the 16S rRNA gene sequence reveales that 23 strains among the 113 bacteria isolated from stem liquid of *P. euphratica* in Mulei Primitive forest belong to Actinobacteria_c, accounting for 20.4%; 49 strains belong to Gammaproteobacteria, accounting for 43.4%; 3 strains belong to Alphaproteobacteria*,* accounting for 2.6%; 38 strains belong to Firmicutes *Bacilli,* accounting for 33.6%. All the 113 strains can be classified into 3 phyla, 4 classes, 9 orders, 17 families and 19 genera, in which the *Bacillus* is the most numerical genus, and the *Brenneria Salicis* is the most dominant species, represented by 22 bacterial isolates (Table 2, Fig. 3).

**Table 2:** Diversity of all isolated specious of different samples. LM:bacteria isolated from stem liquid of *P. euphratica* in Mulei; SM: bacteria isolated from resina on *P. euphratica* in Mulei; LE: bacteria isolated from stem liquid of *P. euphratica* in Ebinur Lake; SE: bacteria isolated from resina on *P. euphratica* in Ebinur Lake

Phyla	Genera	LM	SM	LE	SE	Total
	*Actinotalea*	1				1
	*Agrococcus*	1				1
	*Arthrobacter*	2				2
	*Dietzia*	2	2			4
	*Georgenia*	2		1		3
	*Kocuria*	4	3	3	1	11
*Actinobacteria_c*	*Mycetocola*	5				5
*79 strains*	*Nesterenkonia*	5	23	6	10	44
*Account for 18%*	*Nocardioides*	1				1
	*Microbacterium*		1	1		2
	*Okibacterium*			1		1
	*Brachybacterium*			1		1
	*Sanguibacteraceae*			1		1
	*Paracoccus*	1			1	2
	*Rhizobium*	2		1		3
*Alphaproteobacteria*	*Glycocaulis*				1	1
8 strains	*Nitratireductor*				1	1
Account for 1.8%	*Ensifer*			1		1
	*Eiseniicola*			1		1
*Betaproteobacteria*	*Pseudomonas*	16	1	7	1	25
	*Brenneria*	22				22
	*Enterobacter*		2			2
*Gammaproteobacteria*	*Acinetobacter*			7		7
123 strains	*Halomonas*	11	3	41	12	67
Account for 28.70%	*Enhydrobacter*			1		1
	*Luteimonas*			2		2
	*Bacillus*	25	45	44	36	153
	*Paenibacillus*	1				1
Firmicutes	*Planococcus*	9	17	25	3	54
225 strains	*Sinobaca*	2				2
Account for 51.2%	*Staphylococcus*	1	3			4
	*Anaerobacillus*		4		1	5
	*Chungangia*		1			1
	*Gracilibacillus*		2			2
	*Ornithinibacillus*		2			2
	*Piscibacillus*			1		1
	Total	113	109	145	67	434

The 109 strains isolated from resina on *P. euphratica* in Mulei Primitive forest were classified into 3 phyla (Gammaproteobacteria, Firmicutes, Actinobacteria), 6 orders, 8 families and 14 genera. Of them, 74 isolates belong to Firmicutes *Bacilli*, accounting for 67.8%, and 6 belong to Gammaproteobacteria, accounting for 5.5%. The genus with the highest frequency of isolation is *Bacillus*, with a total of 45 strains, followed by the genus *Nesterenkonia* with 23 strains. The dominant species are the *Bacillus zhangzhouensis* and *Nesterenkonia populi* (Fig. S1)*.*

The 145 bacteria isolated from stem liquid of *P. euphratica* in Ebinur Lake Wetland Nature Reserve are classified as Gammaproteobacteria (40%), Firmicutes *Bacilli* (48%), Actinobacteria_c (9.7%), Alphaproteobacteria (1.4%), Betaproteobacteria (accounting for 0.7%) 3 phyla, 5 classes, 8 orders, 15 families and 19 genera. The predominant genera are *Bacillus* with 44 strains, followed by *Halomonas* with 41 strains. The dominant species are *Bacillus zhangzhouensis* and *Halomonas songnenensis* (Fig. S2)*.*

The 67 strains isolated from resina on *P. euphratica* in Ebinur Lake Wetland Nature Reserve are classified into four groups: Gammaproteobacteria (19.4%), Firmicutes *Bacilli* (59%), Actinobacteria_c (16.4%) and Alphaproteobacteria (4.5%). There are 3 phyla, 4 classes, 6 orders, 8 families and 10 genera. The dominant genus is *Bacillus*, accounting for 53%, and the followed dominant species is *Bacillus zhangzhouensis*, accounting for 17.9%. (Fig. S3).

As we can see, the most numerous genus isolated is *Bacillus* in every sample. And it is found that compared with the stem liquid, more Firmicutes are isolated from resina. Gammaproteobacteria is the second dominant phylum, including Alphaproteobacteria, Betaproteobacteria and Gammaproteobacteria. Among them, Gammaproteobacteria is the most abundant class (125 of the 135 isolates, accounting for 92.6%), and Betaproteobacteria is only separated from the stem liquid of *P. euphratica* in Ebinur Lake. The bacteria isolated from resina on *P. euphratica* in Mulei contains tiny strains belonging to Proteobacteria (5.5%). *Halomonas* is easier found from *P. euphratica* in Ebinur Lake than that in Mulei. Actinobacteria has the least isolates, with the only class Actinobacteria_c. And it can be seen that *P. euphratica* in Mulei contains more Actinobacteria than that in Ebinur Lake (Fig. S4).

### Detection rate of new species

According to the classification of prokaryotic species by Mincheol [31], strains with 16rRNA gene sequence similarity less than 98.65% can be identified as potential new species. The results show that a total of 177 strains are potential new species. Among them, 71 strains belong to *Bacillus*, accounting for 39%; 63 strains belong to Gammaproteobacteria, accounting for 35%. 30 isolated strains from stem liquid of *P. euphratica* in Mulei are potential new species, and the detection rate of new species was 26.5%. And 56 strains isolated from resina, accounting for 51%, are potential new species. 69 isolated strains from stem liquid of *P. euphratica* in Ebinur Lake are potential new species, accounting for 47.5%. 25 strains isolated from resina in Ebinur Lake, accounting for 37.3%, are potential new species.

### Diversity analysis of growth-promoting function of culturable bacteria in P. euphratica

In this study, the isolated stem liquid bacteria and resina bacteria were screened, and 158 functional bacteria with plant growth-promoting potential were obtained. Some of these bacteria have only one function, some have more than two. Specific research results are as shown in Tab. 3.

**Table 3:** The number of strains with growth-promoting function

	Phosphorus solubilizing	Potassium solubilizing	Nitrogen fixation	ferric ammonia salts ultilizing
stem liquid bacteria	34	56	20	86
resina bacteria	27	24	12	65
Total	61	80	32	151

We selected 15 strains with plant growth-promoting potential among them for the next research on the influence of corn germination and seedling growth. The growth-promoting functions of these 15 strains are as shown in Table 4.

**Table 4:** Evaluation plant growth promoting traits of bacteria from *Populus euphratica*

Bacteria coding	phosphorus dissolving	potassium dissolving	nitrogen fixation	ferric ammonia salts ultilizing
BB5-2	-	+	+	+
TC4a	+	-	-	+
BB33-1	+	-	+	-
TC10	+	-	-	+
TA22	-	+	-	+
TC26B	-	-	+	+
BB 11	-	+	+	-
TC28	-	+	-	+
MA27-2	-	+	-	+
MC5	-	+	+	-
MA2	+	-	-	+
MC19A	-	-	-	+
MD2	-	+	-	+
RC1	+	-	+	-
RC6	-	+	+	-

+ is positive, it has relevant ability;—is negative, it does not have relevant ability

### Effects of growth promoting potential of P. euphratica on germination and seedling growth of maize

**Fig. 4:**
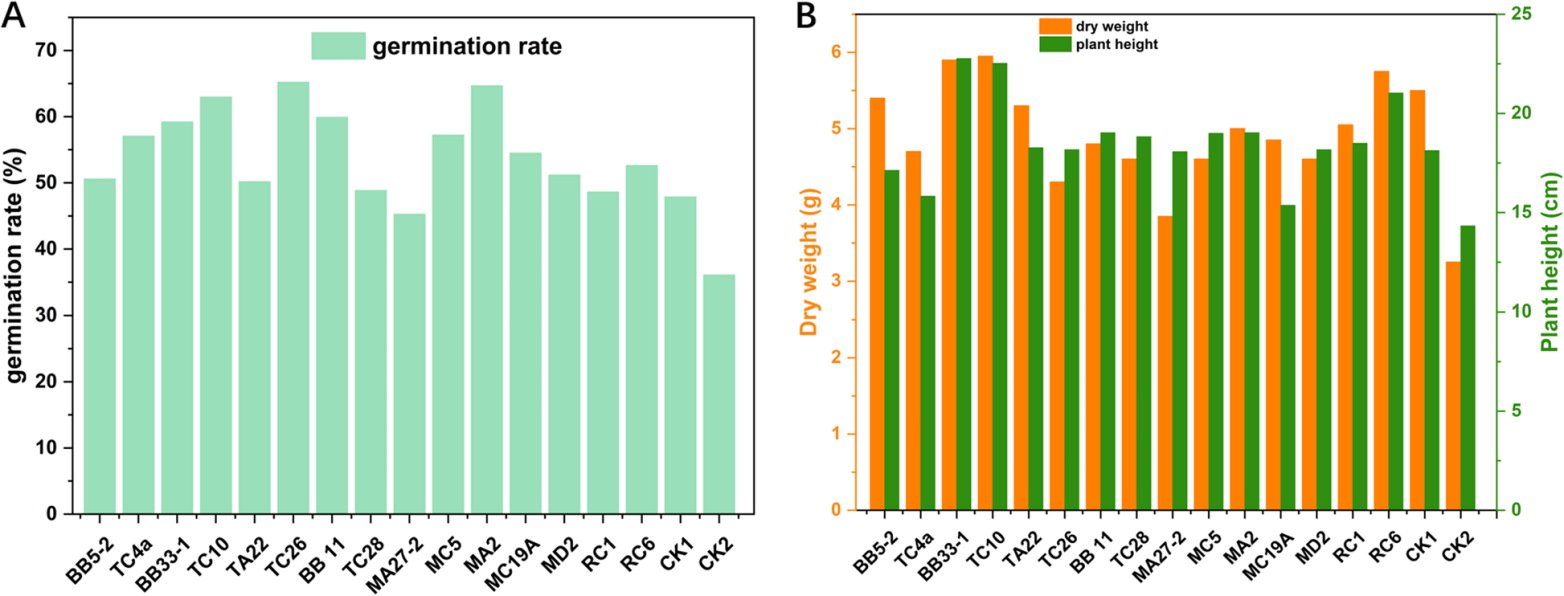
Effects of plant growth promoting bacteria isolated from *P. euphratica* on maize germination rate (**A**), dry weight and plant height **B**

The infected corn seeds began to germinate after 3 days’ culture on the plate, and all seeds were in a stable germination state from the fifth day. The germination rate of CK1 and CK2 and the experimental groups treated with different strains are different to some extent (Fig. 4A). The lowest germination rate is found in the CK2 control group, which is treated with sterile MA media. It is likely that because of the high salt concentration of MA media used in CK2 (NaCl content alone is nearly 20 g/L), many corn seeds did not sprout. Among the 15 experimental groups, except TC28, MA27-2 and RC6, the germination rates of other treatment groups are higher than that of CK1. The experimental groups with significantly higher average germination rates are BB33-1, TC10, TC26, BB11, MC5 and MA2.

Generally speaking, plant height is a morphological indicator, which can well reflect the good growth of maize plants [32]. Moreover, the absorption of many nutrients, such as nitrogen, has a great influence on plant height [33]. The biomass of fresh weight and dry weight reflects the accumulation of photosynthetic products of maize, which is a measure of plant productivity (Fig. 4B). After planting maize buds in pots for 20 days, the plant height and dry weight of corn seedlings were measured to further confirm the growth-promoting ability of functional bacteria with growth-promoting potential. The results show that the plant height and dry weight of corn seedlings in the CK2 control group are still very low. The average dry weight of corn seedlings in the CK1 control group is 5.5 g, while that in BB33-1, TC10 and RC6 are heavier than that in CK1. The average dry weight of maize seedlings in other experimental groups is close to or lower than CK1. In terms of corn seedling height, the average seedling height of CK1 is 18.1 cm, the average seedling height of BB33-1, TC10 and RC6 in the experimental group is higher than that in CK1, and the average seedling height of other treatment groups is close to or lower than CK1.

Based on the above research results, strains BB33-1, TC10 and RC6 had certain growth-promoting abilities, and their genome analysis and physiological and biochemical studies were carried out.

### Description of growth-promoting bacteria

**Fig. 5:**
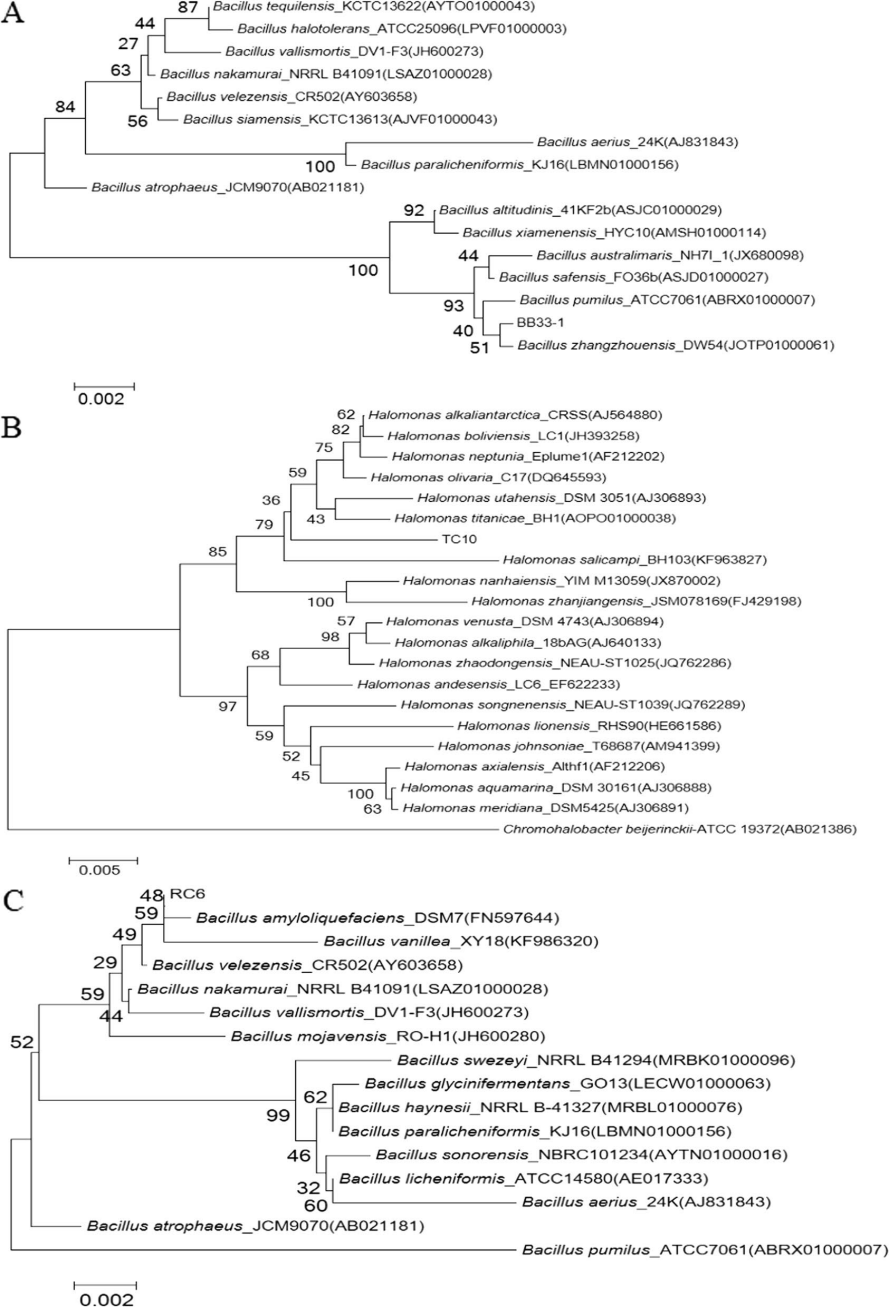
Neighbor-joining phylogenetic tree based on the 16S rRNA gene sequences of strain BB33-1 (**A**), TC10 (**B**) and RC6 (**C**), and representatives of related taxa

Phylogenetic analysis of strain BB33-1 based on 16S rRNA gene sequences indicates that the strain belonged to the genus *Bacillus* and exhibited 16S rRNA gene sequence similarities to the strains *Bacillus Zhangzhouensis* DW5-4 T of 99.93%. The phylogenetic tree (Fig. 5A) based on the phylogenomic analysis was reconstructed and BB33-1 also formed a cluster with *Bacillus Zhangzhouensis* DW5-4 T. Using the same method to analyze RC6 (Fig. 5B), it can be concluded that strain RC6 belongs to the genus *Bacillus* and showed the highest 16S rRNA gene sequence similarity of 99.71% to *Bacillus amyloliquefaciens* DSM 7_FN597644. As for TC10 (Fig. 5C), the sequences similarity with *Halomonas olivaria* C17_DQ645593T is 98.39%, And phylogenetic tree analysis shows that TC10 belong to an independent clade and may be a potential new species.

The basic physiological and biochemical characteristics of BB33-1, TC10 and RC6 are shown in Table 5. In addition, the fermentation of strain BB33-1 was positive for alkaline phosphatase, acid phosphatase, esterase (C4), esterase lipase, mannitol, inositol, sucrose, amygdaloside and arabinose. Urease, tryptophan deaminase, indole production, saccharide, sorbitol and rhamnose fermentation were negative. TC10 can produce acid from glucose, D-galactose, D-ribose, sucrose and trehalose, and can use acetic acid, lactic acid, citric acid and succinic acid as the carbon source. Accurate classification and evolutionary status require further multiphase taxonomic identification. RC6 is negative for starch and nitrate reduction. Lactose and sugar can be used to produce acid, but not pine, trehalose, honey, or d-arabinose to produce acid.

**Table 5:** Basic physiological and biochemical characteristics of the 3 strains

	BB33-1	TC10	RC6
Gram staining	** + **	** + **	** + **
Colony color	yellow	dark yellow	milky white
Growth temperature	8–45 °C,	4–50 °C	4–55 °C
pH	5–11	5–10	4.5–9
salt concentration	0–12%	0–25%	0–14%
Catalase	** + **	** + **	** + **
Oxidase	** + **	** + **	**-**
V-P	** + **	**-**	**-**
Glucose fermentation	** + **	** + **	** + **

+ is positive;—is nagetive

### Genome sequencing and analysis

The final assembly of BB33-1 consists of a 3,607,844-bp chromosome with GC contents of 42%. The pipeline detects 3742 protein-coding genes, 46 transfer RNA genes, and 8 ribosomal RNA genes. The final assembly of RC-6 consists of a 3,893,161-bp chromosome with GC contents of 46%. The pipeline detects 3891 protein-coding genes, 44 tRNA genes, and 10 rRNA genes. The final assembly of TC-10 consists of a 4,489,612-bp chromosome with GC contents of 54%. The pipeline detects 4297 protein-coding genes, 53 tRNA genes, and 5 rRNA genes (Table 6).

**Table 6:** Genome-wide splicing and gene element prediction

Class	BB33-1	RC-6	TC-10
Size(base)	3,607,844	3,893,161	4,489,612
G + C content (%)	42	46	54
Protein Coding Genes	3742	3891	4297
Min length(base)	45	45	74
Max length(base)	10,134	16,299	8703
Average length(base)	850.85	885.03	931.76
Total coding gene(base)	3,183,881	3,443,650	4,003,777
Coding ratio (%)	88.25	88.4558	89.18
tRNA	46	44	53
rRNA	8	10	5

**Fig. 6:**
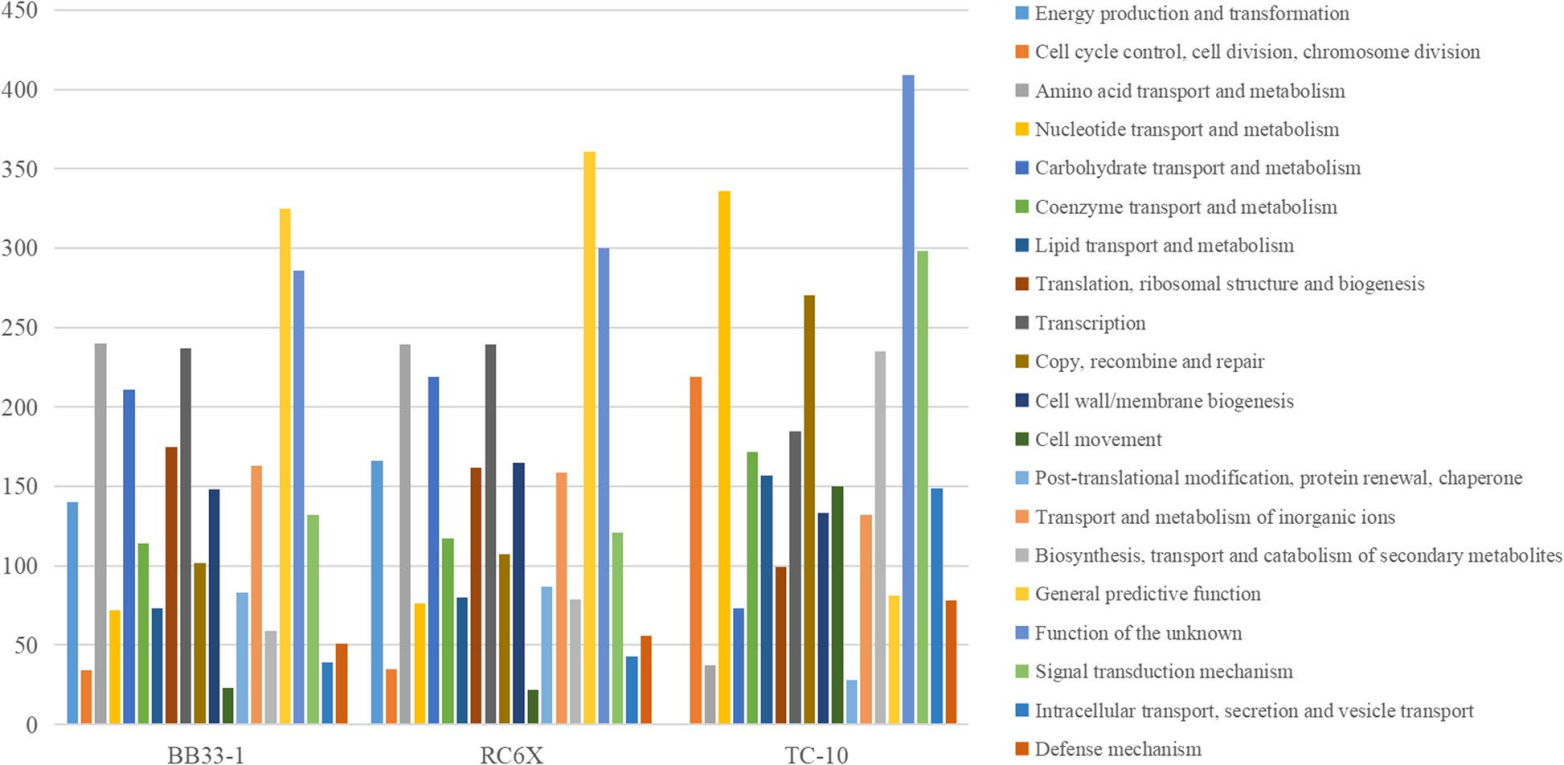
COG functional classification

The COG annotation results are shown in Fig. 6. The protein-coding genes are divided into 10 categories, among which unknown functional genes account for a large proportion, which need to be further explored and explored. The number of genes with different functions is also different, but the abundance of genes belonging to the same genus BB33-1 and RC6 is consistent. KEGG annotation of the genomes of three growth-promoting strains shows that 20 genes related to the replication and metabolic function of plant cells can be mapped to five metabolic pathways (Table 7).

**Table 7:** Metabolic pathways of three growth-promoting bacteria

Pathway name	BB33-1	RC6X	TC10
Pentose phosphate pathway	25	25	21
Methane metabolism	22	22	24
Nitrogen metabolism	8	18	16
Thiamine metabolism	17	16	13
Glycolysis/Gluconeogenesis	43	36	26
Pantothenic acid and CoA biosynthesis	19	20	24
Biosynthesis of terpenoid skeletons	14	15	15
Biosynthesis of zeatin	1	1	1
TCA cycle	27	23	22
photosynthesis	8	8	8
carbon metabolism	95	89	98
Biosynthesis of tetracycline	5	5	4
Hif-1 signaling pathway	7	5	4
Biotin metabolism	10	13	15
Carbon sequestration pathways in prokaryotes	29	27	34
Plant pathogens interaction	4	5	8
Folic acid biosynthesis	20	19	15
PTS	33	20	4
Carbon fixation in photosynthetic organisms	11	12	14
Porphyrin and chlorophyll metabolism	16	18	36

For further accurate secondary metabolism BGC (SMBGC) mining analysis, 14 SMBGCs of strain BB33-1, 22 SMBGCs of strain RG6 and 6 SMBGCs of strain TC-10 were proposed by using anti SMASH 5.0 [34] (Table S2-4).

BLASTN analysis shows that the sequence similarity between the NRPS gene cluster 1 of strain BB33-1 and the bacillibactin biosynthetic gene cluster of *Bacillus subtilis* Subsp. subtilis str. 168 (NCBI accession number: AL009126.3) is 53% [35, 36], while it has a higher sequence similarity with *Bacillus velezensis* FZB42 bacillibactin biosynthetic gene cluster (NCBI accession number: CP000560.1) [37], which is 100%, so Cluster 1 of strain BB33-1 is predicted to be involved in the synthesis of compounds similar to bacillibactin. Cluster 4 of BB33-1 has an 85% sequence similarity with *Bacillus velezensis* FZB42 bacilysin biosynthetic gene Cluster (NCBI accession number: CP000560.1) [38–40], predicting that Cluster 4 might be involved in the synthesis of bacilysin compounds. Terpene gene cluster of strain BB33-1 (Cluster 11) and Carotenoid biosynthetic gene Cluster of *Halobacillus Halophilus* DSM 2266 (NCBI accession number: FJ040212.1) is the most similar (50%) [41], which is predicted to be involved in the biosynthesis of Terpene class compounds. Cluster 13 of BB33-1 and *Bacillus velezensis* FZB42 fengycin biosynthetic gene cluster (NCBI accession number: CP000560.1) are highly similar (53%), so Cluster 13 may be involved in the biosynthesis of this NRP class compound [42, 43].

As for strain RC-6, it has some gene clusters that may be involved in the biosynthesis of Bacillibactin, Bacilysin and fengycin. The sequence similarity between transat-pks gene Cluster (Cluster 8) and *Bacillus velezensis* FZB42 macrolactin H gene cluster (NCBI accession number: AJ634061.2) is 100% [44, 45]. Cluster 8 is predicted to be involved in the synthesis of macrolactin H compounds. Bacillaene biosynthetic gene Cluster with *Bacillus velezensis* FZB42 (NCBI accession number: AJ634060.2) sequence similarity is 100% [46, 47]. Cluster 9 is predicted to be involved in the synthesis of bacillaene.

Sequence similarity between Cluster 2 of STRAIN TC-10 and *Methylomicrobium Kenyense* ectoine gene Cluster (NCBI Number: DQ238213.1) is 75% [48] based on BLASTN, predicting Cluster 2 may be involved in the synthesis of ectoine compounds.

## Discussions

### Culturable bacterial species diversity

According to 16S rRNA sequence analysis and phylogenetic analysis, endophytic bacteria and resina bacteria isolated from *P. euphratica* stem fluid and resina in Mulei and Ebinur Lake belong to Actinobacteria_c, Alphaproteobacteria, Gammaproteobacteria, Firmicutes, respectively. But there is another endophytic bacterium isolated from *P. euphratica* stem fluid in Ebinur Lake belonging to Betaproteobacteria (MC8, the similar strain was *Eiseniicola Composti*, and the sequence similarity is 98.1%). Compared with the endophytic bacteria community structure of *P. euphratica* in the Primitive *P. euphratica* forest in Tarim River, Betaproteobacteria and Bacteroidete are not isolated from the *P. euphratica* in Mulei [49]. Bacteroidetes are not isolated from *P. euphratica* in Ebinur Lake. However, the proportion of Betaproteobacteria and Bacteroidete in the endophytic bacteria community of *P. euphratica* in Tarim River is also not high, which is 0.34% and 0.50% respectively. Therefore, these two phyla are considered to be the vulnerable groups in *P. euphratica* culturable bacteria. The main forest area in the middle reaches of Tarim River is sparsely populated, which is less affected by human production and living activities, and maintains the original ecological state. Therefore, the community structure of endophytic bacteria in *P. euphratica* in Tarim shows its inherent richness, with relatively uniform phylogeny and relatively stable community structure. However, the area near the *P. euphratica* forest in Ebinur Lake is the floodplain area, which is close to the water source and water plants, and close to the nearby farmland and residential areas, so grazing has a certain impact on the area. The primeval *P. euphratica* forest in Mulei is in a state of decline, with fewer young *P. euphratica* alive and most old ones withering, which is also affected by grazing. Therefore, culturable bacteria belonging to Betaproteobacteria and Bacteroidete may be replaced or cleared. It is noteworthy that P*seudomonas Populi* was isolated in PEA media, and there are two strains, PC17 and PC18, with sequence similarities of 98.9% and 99.6%, respectively. When the author participated in the diversity study of endophytic bacteria of *P. euphratica* in Tarim, an endophytic strain of *P. euphratica* was isolated and described as a new species through the multiphase taxonomic identification method and named as *Pseudomonas Populi* [50]. The endophytic bacteria which were isolated from both Tarim *P. euphratica* forest and *P. euphratica* forest in Ebinur Lake, indicate that there is a special relationship between these species and *P. euphratica*.

The detection rate of potential new strains refers to the ratio of the number of potential new strains to the number of isolated strains in the community to which they belong. It is relatively simple to judge whether the community structure has been invaded and its change degree by the detection rate of potential new species. However, it is only suitable for the analysis of communities with more original new strains, but it can’t be used for the analysis of communities with few or no original new strains. In this study, the new species detection rate in Ebinur Lake (47.6% of stem liquid bacteria and 38.8% of resina bacteria) is higher than that in Mulei (26.50% of stem liquid bacteria and 51.4% of resina bacteria), which may indicate that the original state of Ebinur Lake is better preserved. However, the relationship between endophytic bacteria community structure and the detection rate of new species of *P. euphratica* needs further study and analysis, and so does resina bacteria.

In addition, the culture method is an important factor affecting the diversity of endophytic bacteria, and it also affects the diversity of isolated bacteria. In this study, the dilution coating method was used to separate and screen the bacteria, which reduced the interference of the dominant bacteria to the slow-growing bacteria. Endophytic bacteria of plants are fixed in plants for a long time so reasonable screening conditions are designed according to the properties of plant samples, such as the growth environment of host plants, and the metabolic characteristics of bacteria to be isolated [3]. Culture media that simulate the internal environment of plants as much as possible will isolate and obtain more endophytic bacteria resources. Therefore, we used the self-designed PEA media, which is added with *P. euphratica* trunk extract. And the dominance index of self-made PEA media is 0.408, which is second only to R2A media. *P. euphratica* has strong characteristics of drought tolerance and salinity tolerance. The salt concentration of *P. euphratica* internal fluid is very high. Therefore, we used five media with different salt concentrations. And it can be seen that the species and proportion of genera isolated from different media are different. It is worth noting that *Halomonas,* the genus massively isolated, cannot be isolated from the BPA media. *Halomonas* is an important representative of moderate halophilic bacteria, but BPA media do not contain any salt. It may be the reason that no *Halomonas* can be isolated from the BPA media. Strains belonging to *Bacillus, Kocuria, Nesterenkonia, Pseudomonas* and *Planococcus* grow on all five kinds of media, which means these genera adapt to different salt concentrations of 0–30%. In addition, there are a lot of bacteria belonging to *Bacillus* isolated from every medium, indicating that *Bacillus* is highly adaptable.

However, the proportion of culturable bacteria remains low, and even fewer bacteria can be cultivated in the laboratory due to the constraints of experimental conditions. The number of strains isolated from *P. euphratica* internal liquid and resina at different sampling sites is less, with an average of 100 strains around, and they distribute in only 36 genera. This is far from the abundance and diversity of bacteria contained in *P. euphratica* forests. Therefore, great improvements are needed to study endophytic bacteria diversity in future studies.

### Growth-promoting function of culturable bacteria

In this study, 434 strains isolated from *P. euphratica* were screened, and 158 functional bacteria with plant growth-promoting potential were obtained. 15 strains were selected to treat corn seeds with their bacterial solution, and then used distilled water and sterile MA media as a control group to accelerate germination and pot cultivation. The results show that the germination rate, seedling height and dry weight of corn seeds treated with these 15 functional bacteria are higher than those of the CK2 control group treated with sterile MA media. The salt content of MA media is relatively high. According to studies, high salinity affects the osmotic metabolism of plants and the normal physiology and biochemistry of cells [51], so it inhibits the germination of corn seeds to a certain extent. Under the condition of salt stress, the seed germination rate, seedling height and seedling dry weight of the treatment group are close to or greater than those of the control group treated with distilled water. These results indicate that these growth-promoting functional bacteria also enhance resistance.

Endophytic bacteria in different environments and at different growth stages have different bacterial groups [52]. If the selected microorganisms cannot colonize and reproduce in the plant, the functions and effects of the microorganisms cannot be fully played, thus affecting the treatment effect. Some endophytic bacteria have the potential to promote plants growth and improve plants resistance, but the exact mechanism, and the relationship between participation ways are not clear. Different endophytic bacteria on the same kind of plants, or the same endophytic bacteria on different plants may be disparate, which needs further researches.

In this study, strains BB33-1 and RC6, with obvious growth-promoting effects, both belong to *Bacillus* (*Bacillus Zhangzhouensis* and *Bacillus siamensis*). In this study, it is found that BB33-1 belonging to *Bacillus* has phosphorus solubilization ability and nitrogen fixation ability, while RC6 has potassium solubilization ability and nitrogen fixation ability. *Bacillus* is one of the dominant species in soil micro-ecology [53] and can form spores with strong stress resistance, which is beneficial to its survival and colonization in the production and utilization of bio-organic fertilizer and soil micro-ecological environment [54]. *Bacillus* has been widely studied and used in various fields because of its characteristics of strong survival ability, high reproductive capacity and convenient production [55, 56]. *Bacillus* can promote plants growth by synthesizing a variety of different growth hormones by itself, and it can produce obvious physiological effects at an extremely low concentration or in an extremely hot condition [57]. Therefore, compared with other microorganisms, it has incomparable advantages, such as colonization and reproduction in the stem, and has a wide application prospect. However, due to the limitations of ecological adaptability and environmental resistance, the growth of *Bacillus* is unstable, and the effective bactericidal substances secreted by *Bacillus* may also be degraded, which would lead to an unstable control effect [58]. Thus, there is still an urgent need for *bacillus* strains with efficient and stable growth-promoting functions [59]. At present, it is known that 239 *Bacillus* strains have completed the whole gene sequencing (https://www.ncbi.nlm.nih.gov/genome/?term=bacillus). The complete genomic data of each strain provide a theoretical basis for the in-depth study of microorganisms [60]. The first complete genome sequence of Samaria fonticola DSM 4576 T, showing PGPR characteristics, can produce indole-3-acetic acid and dissolve inorganic phosphate [61]. *Bacillus velezensis* S3-1, isolated from cucumber rhizosphere soil, has the characteristics of inhibiting plant pathogens [62]. Its genomic information shows that it contains 57 biosynthetic gene clusters encoding bactericide, surfactant and ubiquitin. It provides useful information for the molecular mechanism behind the antifungal effect.

The strain TC10 with phosphorus solubilization ability and ferric ammonia salt utilizing ability belongs to *Halomonas*, and the growth-promoting effect is also obvious. The similarity between strain TC10 and *Halomonas olivaria* C17_DQ645593T sequence of *Halomonas olivaria* is 98.39%, and the phenotypic, physiological and biochemical characteristics are significantly different [63]. *Halomonas* is an important representative of moderate halophilic bacteria, which is gram-negative, aerobic and rod-like, and mainly distributed in salt lakes, salt farms, oceans, or extreme marine environments [64]. In addition to its strong adaptability due to its unique structural characteristics and special physiological mechanism, it also has some physiological characteristics that other microorganisms do not have, such as the ability to degrade aromatic compounds, to achieve denitrification [65], to produce exopolysaccharides [66] and polyhydroxybutyrate [67], and so on. Due to their low requirements for nutrition, they are easier to adapt to the environment and grow and reproduce in a high-salt environment, thus can minimize the pollution of other fungi in the fermentation process. And a variety of organics can be used as carbon and nitrogen sources. In recent years, there have been a lot of studies on *Halomonas*, such as the discovery of a new species of *Halomonas* [68, 69], molecular identification of new species [70], detection of physiological and biochemical activities of related products [71][72] and analysis of salt-tolerant genes and so on. However, there are few reports on the application of *Halomonas*, and no research reports its growth-promoting function. Some gene characteristics and expression characteristics of strain TC10 can be considered as a new species, and its precise classification and evolutionary status, and mechanism of promoting plants growth need further study.

This experiment reports the whole genome information of three strains of growth-promoting bacteria from *P. euphratica*. The statistics and quality analysis of the second-generation sequencing data of this genome show that the sequencing quality is high and the data are reliable. Bacterial whole genome sequencing analysis can more accurately excavate the excellent genes unique to bacterial strains, which is conducive to further exploring their regulatory mechanism. The discovery of these functional genes will lay a theoretical foundation for the later study of promoting the mechanism of strains.

## Supplementary Information

**Additional file 1: Table S1. **Comparison of different media used for isolation.** Table S2.** Gene cluster of secondary metabolites of strain BB33-1 predicted by antiSMASH.** Table S3.** Gene cluster of secondary metabolites of strain RC-6 predicted by antiSMASH.** Table S4.** Gene cluster of secondary metabolites of strainTC-10 predicted by antiSMASH.** Fig. S1.** Neighbour-joining tree of culturable bacteria isolated from sap of P. euphratica at Aibi Lake. Bootstrap values are indicated at the nodes.** Fig. S2.** Neighbour-joining tree of culturable bacteria isolated from storage liquid of P. euphratica at Aibi Lake. Bootstrap values are indicated at the nodes.** Fig. S3.** Neighbour-joining tree of culturable bacteria isolated from sap of P. euphratica at Mori original P. euphratica forest . Bootstrap values are indicated at the nodes.** Fig. S4.** Neighbour-joining tree of culturable bacteria isolated from storage liquid of P. euphratica at Mori original P. euphratica forest . Bootstrap values are indicated at the nodes.

## Data Availability

All data generated or analyzed during this study are included in this published article [and its supplementary information files]. The raw reads of pyrosequencing data have been deposited into the NCBI Sequence Read Archive (SRA) database repository, [https://www.ncbi.nlm.nih.gov/sra] and [Accession Number: JALPZO000000000,JALPZN000000000].
