# Supplementary material for: Culturable bacteria diversity in stem liquid and resina from Populus euphratica and screening of plant growth-promoting bacteria
Source: BMC Microbiol. 2022 Dec 29;22:322. doi: 10.1186/s12866-022-02731-7 (PMC9798617; doi:10.1186/s12866-022-02731-7)
Supplement: Supplementary file 1 — Additional file 1: Table S1. Comparison of different media used for isolation. Table S2. Gene cluster of secondary metabolites of strain BB33-1 predicted by antiSMASH. Table S3. Gene cluster of secondary metabolites of strain RC-6 predicted by antiSMASH. Table S4. Gene cluster of secondary metabolites of strainTC-10 predicted by antiSMASH. Fig. S1. Neighbour-joining tree of culturable bacteria isolated from sap of P. euphratica at Aibi Lake. Bootstrap values are indicated at the nodes. Fig. S2. Neighbour-joining tree of culturable bacteria isolated from storage liquid of P. euphratica at Aibi Lake. Bootstrap values are indicated at the nodes. Fig. S3. Neighbour-joining tree of culturable bacteria isolated from sap of P. euphratica at Mori original P. euphratica forest . Bootstrap values are indicated at the nodes. Fig. S4. Neighbour-joining tree of culturable bacteria isolated from storage liquid of P. euphratica at Mori original P. euphratica forest . Bootstrap values are indicated at the nodes. [file 12866_2022_2731_MOESM1_ESM.docx]

**Tab.S1** Comparison of different media used for isolation

| Chemical composition | LB | BPA | PEA | MA | TSB | R2A |
| --- | --- | --- | --- | --- | --- | --- |
| PH | 7.0-7.5 | 7.0-7.5 | 7.0-7.5 | 7.5-8.0 | 8.0-8.5 | 7.5-8.0 |
| Peptone | 10g | 5g | 2g | 2.0g |  | 0.25g |
| Yeast extract powder | 5g | 1g | 1g | 0.3g |  | 0.5g |
| Sodium chloride | 20g |  | 5g | 20g | 30g | 10g |
| Sodium sulfate |  |  |  | 1.08g |  |  |
| Eerric citrate |  |  |  | 0.03 |  |  |
| Magnesium chloride |  |  |  | 2.9g |  |  |
| Calcium chloride |  |  |  | 0.6 |  |  |
| Potassium chloride |  |  |  | 0.18g |  |  |
| Mother liquor |  |  |  | 3.5ml |  |  |
| Tryptone |  |  |  |  |  | 0.25g |
| Soya peptone |  |  |  |  | 1.5g |  |
| Casein peptone |  |  |  |  | 8.5g | 0.5g |
| Potassium phosphate  monobasic |  |  |  |  | 1.25 |  |
| Glucose |  |  | 1g |  |  | 0.5g |
| Soluble starch |  |  |  |  |  | 0.5g |
| Potassium phosphate  monobasic dehydrate |  |  |  |  |  | 0.3g |
| Magnesium sulfate |  |  |  |  |  | 0.1g |
| Sodium pyruvate |  |  |  |  |  | 0.3g |
| Beef extract |  | 3g |  |  |  |  |
| Populus  extract solution |  |  | 10ml |  |  |  |
| Water | 1L | 1L | 1L | 1L | 1L |  |
| Agar powder | 20g | 20g | 20g | 18g | 18g | 20g |


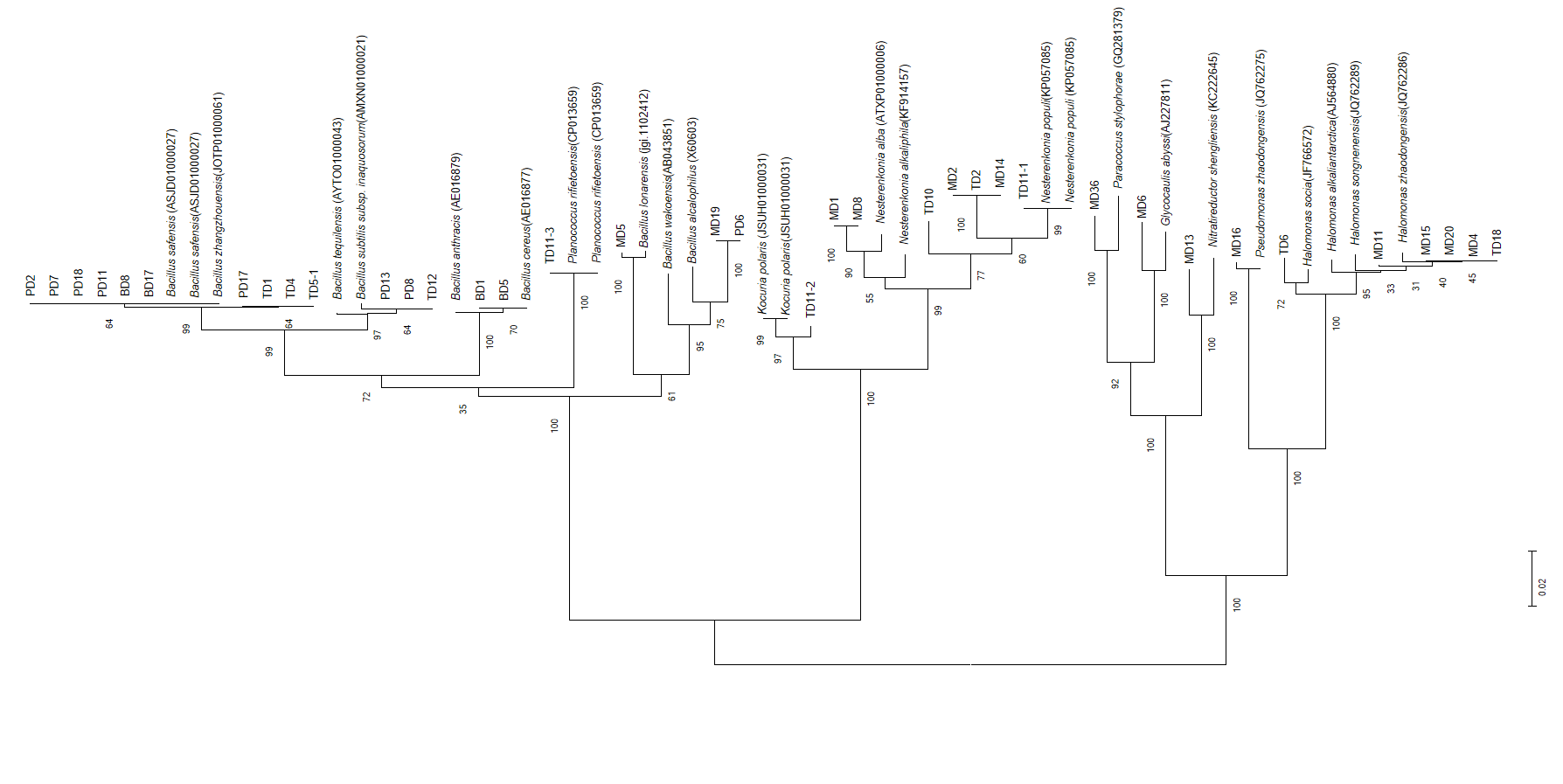


**Fig. S1** Neighbour-joining tree of culturable bacteria isolated from sap of *P. euphratica* at Aibi Lake. Bootstrap values are indicated at the nodes.


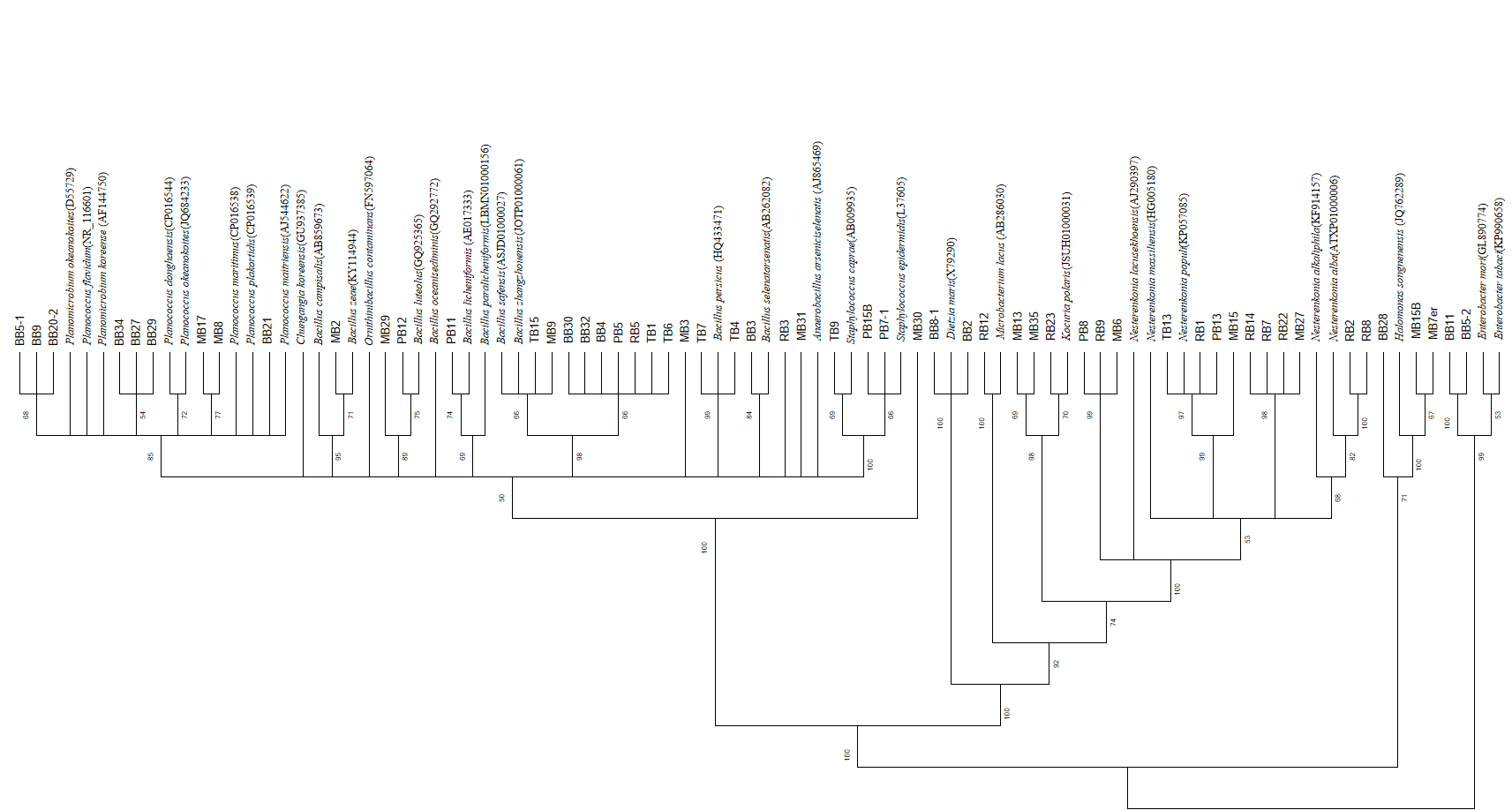


**Fig. S2** Neighbour-joining tree of culturable bacteria isolated from storage liquid of *P. euphratica* at Aibi Lake. Bootstrap values are indicated at the nodes.


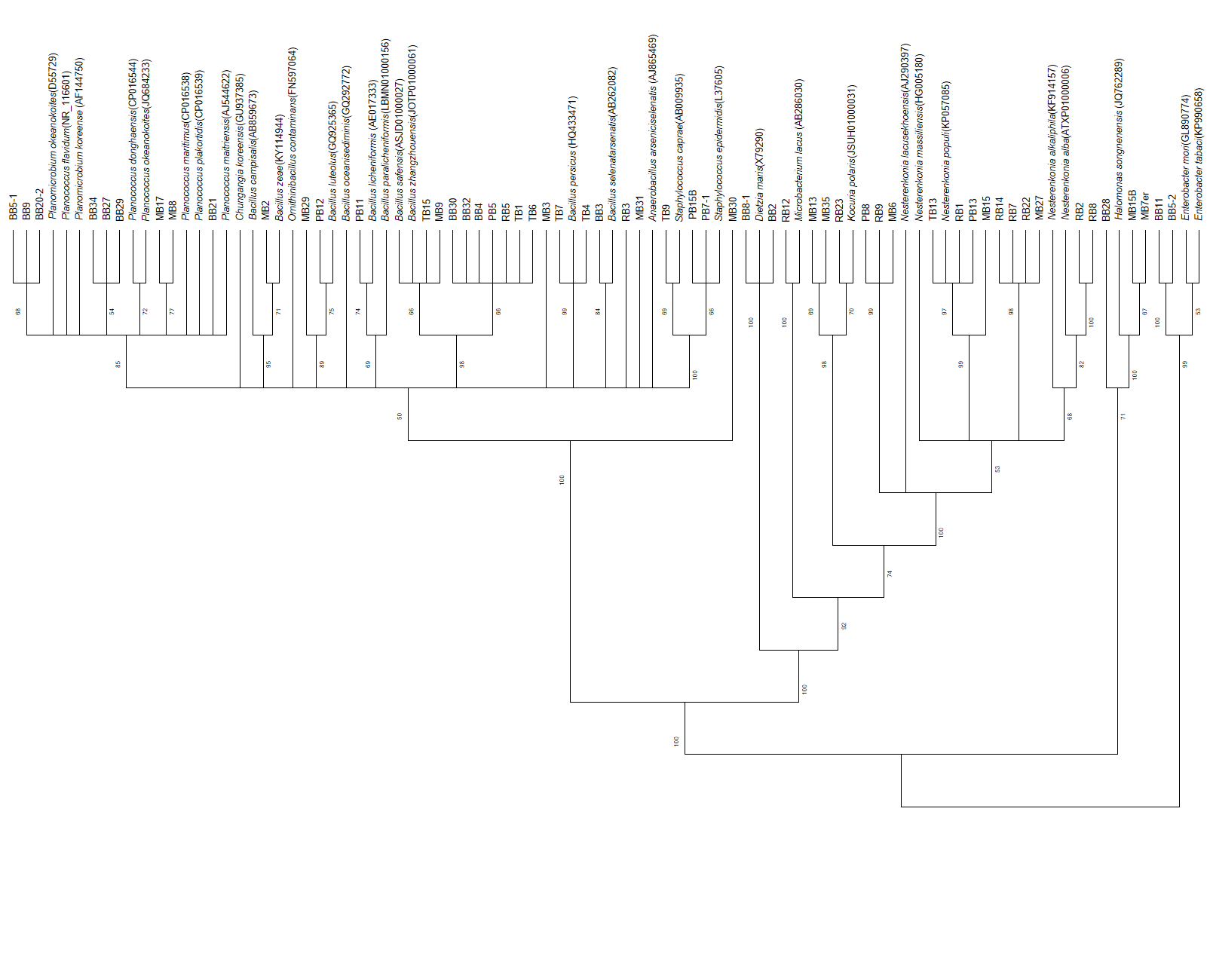


**Fig. S3** Neighbour-joining tree of culturable bacteria isolated from sap of P. euphratica at Mori original *P. euphratica* forest . Bootstrap values are indicated at the nodes.


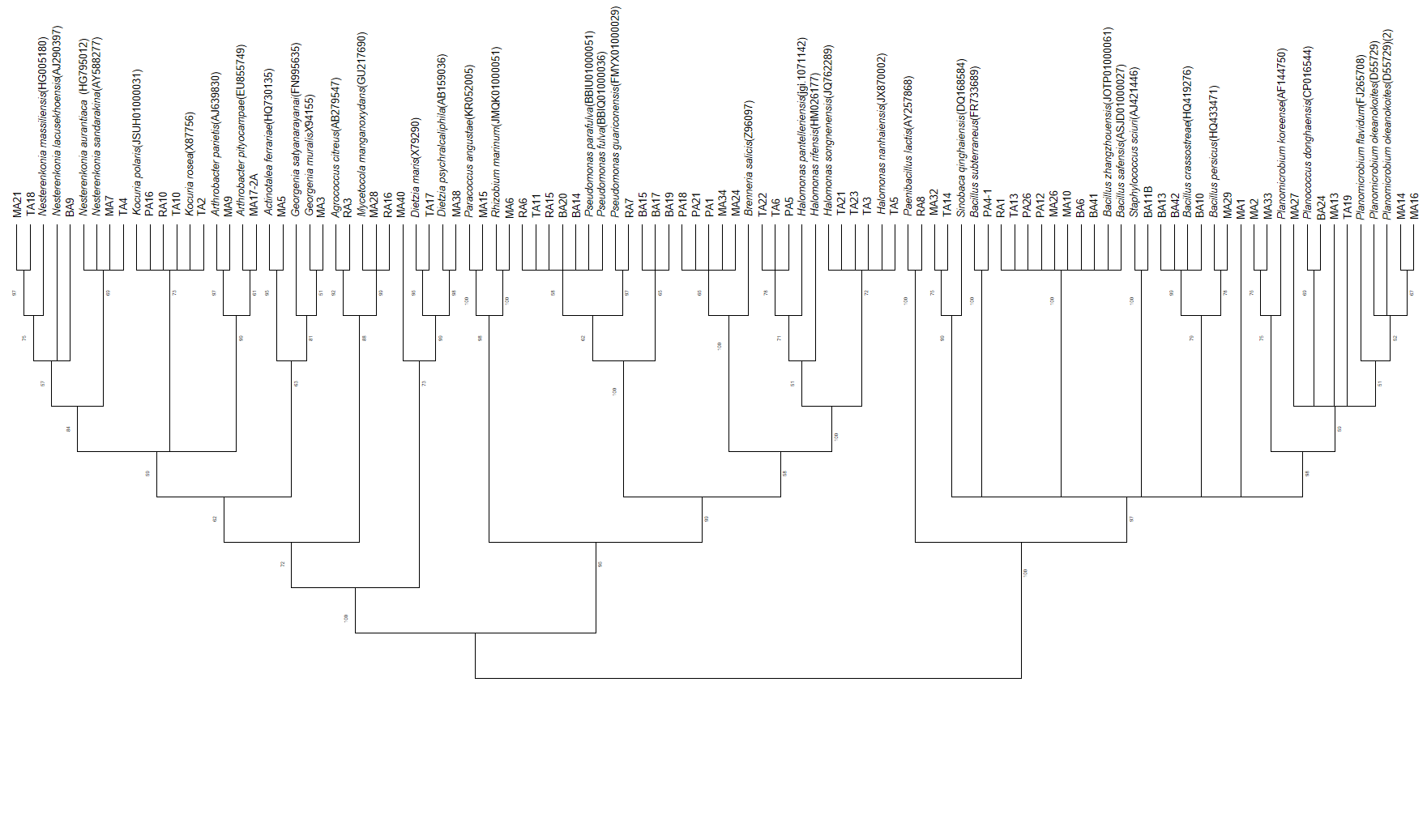


**Fig. S4** Neighbour-joining tree of culturable bacteria isolated from storage liquid of *P. euphratica* at Mori original *P. euphratica* forest . Bootstrap values are indicated at the nodes.

**Tab. S2** Gene cluster of secondary metabolites of strain BB33-1 predicted by antiSMASH

| Cluster | Type | Length (bp) | The Closest BGC | Similarity |
| --- | --- | --- | --- | --- |
| Region 1 | [NRPS](https://docs.antismash.secondarymetabolites.org/glossary/#nrps) | 7766 | [bacillibactin](https://mibig.secondarymetabolites.org/go/BGC0000309/1) | 53% |
| Region 2 | [NRPS](https://docs.antismash.secondarymetabolites.org/glossary/#nrps) | 80634 | [surfactin](https://mibig.secondarymetabolites.org/go/BGC0000433/1) | 43% |
| Region 3 | [sactipeptide](https://docs.antismash.secondarymetabolites.org/glossary/#sactipeptide),[ranthipeptide](https://docs.antismash.secondarymetabolites.org/glossary/#ranthipeptide) | 24378 | [sporulation killing factor](https://mibig.secondarymetabolites.org/go/BGC0000601/1) | 85% |
| Region 4 | [other](https://docs.antismash.secondarymetabolites.org/glossary/#other) | 40222 | [bacilysin](https://mibig.secondarymetabolites.org/go/BGC0001184/1) | 85% |
| Region 5 | [betalactone](https://docs.antismash.secondarymetabolites.org/glossary/#betalactone) | 12357 | - | - |
| Region 6 | [RiPP-like](https://docs.antismash.secondarymetabolites.org/glossary/#ripp-like) | 40716 | - | - |
| Region 7 | [terpene](https://docs.antismash.secondarymetabolites.org/glossary/#terpene) | 21825 | - | - |
| Region 8 | [NRPS](https://docs.antismash.secondarymetabolites.org/glossary/#nrps),[T1PKS](https://docs.antismash.secondarymetabolites.org/glossary/#t1pks) | 28973 | [paenilamicin](https://mibig.secondarymetabolites.org/go/BGC0001033/1) | 28% |
| Region 9 | [NRPS](https://docs.antismash.secondarymetabolites.org/glossary/#nrps) | 73331 | [lichenysin](https://mibig.secondarymetabolites.org/go/BGC0000381/1) | 50% |
| Region 10 | [RRE-containing](https://docs.antismash.secondarymetabolites.org/glossary/#rre-containing) | 60658 | - | - |
| Region 11 | [terpene](https://docs.antismash.secondarymetabolites.org/glossary/#terpene),[siderophore](https://docs.antismash.secondarymetabolites.org/glossary/#siderophore) | 19926 | [carotenoid](https://mibig.secondarymetabolites.org/go/BGC0000645/1) | 50% |
| Region 12 | [NRPS](https://docs.antismash.secondarymetabolites.org/glossary/#nrps) | 10630 | [surfactin](https://mibig.secondarymetabolites.org/go/BGC0000433/1) | 8% |
| Region 13 | [betalactone](https://docs.antismash.secondarymetabolites.org/glossary/#betalactone) | 22496 | [fengycin](https://mibig.secondarymetabolites.org/go/BGC0001095/1) | 53% |
| Region 14 | [T3PKS](https://docs.antismash.secondarymetabolites.org/glossary/#t3pks) | 37860 | - | - |

**Tab. S3** Gene cluster of secondary metabolites of strain RC-6 predicted by antiSMASH

| Cluster | Type | Length (bp) | The Closest BGC | Similarity |
| --- | --- | --- | --- | --- |
| Region 1 | [transAT-PKS-like](https://docs.antismash.secondarymetabolites.org/glossary/#transat-pks-like) | 24276 | [difficidin](https://mibig.secondarymetabolites.org/go/BGC0000176/1) | 26% |
| Region 2 | [NRPS](https://docs.antismash.secondarymetabolites.org/glossary/#nrps) | 13068 | [fengycin](https://mibig.secondarymetabolites.org/go/BGC0001095/1) | 20% |
| Region 3 | [NRPS](https://docs.antismash.secondarymetabolites.org/glossary/#nrps) | 10155 | [fengycin](https://mibig.secondarymetabolites.org/go/BGC0001095/1) | 13% |
| Region 4 | [NRPS](https://docs.antismash.secondarymetabolites.org/glossary/#nrps) | 9782 | [surfactin](https://mibig.secondarymetabolites.org/go/BGC0000433/1) | 8% |
| Region 5 | [other](https://docs.antismash.secondarymetabolites.org/glossary/#other) | 41418 | [bacilysin](https://mibig.secondarymetabolites.org/go/BGC0001184/1) | 100% |
| Region 6 | [RiPP-like](https://docs.antismash.secondarymetabolites.org/glossary/#ripp-like),[NRPS](https://docs.antismash.secondarymetabolites.org/glossary/#nrps) | 51791 | [bacillibactin](https://mibig.secondarymetabolites.org/go/BGC0000309/1) | 100% |
| Region 7 | [lanthipeptide-class-ii](https://docs.antismash.secondarymetabolites.org/glossary/#lanthipeptide-class-ii) | 28888 | - | - |
| Region 8 | [transAT-PKS](https://docs.antismash.secondarymetabolites.org/glossary/#transat-pks) | 87835 | macrolactin H | 100% |
| Region 9 | [transAT-PKS](https://docs.antismash.secondarymetabolites.org/glossary/#transat-pks),[T3PKS](https://docs.antismash.secondarymetabolites.org/glossary/#t3pks),[NRPS](https://docs.antismash.secondarymetabolites.org/glossary/#nrps) | 100565 | [bacillaene](https://mibig.secondarymetabolites.org/go/BGC0001089/1) | 100% |
| Region 10 | [NRPS](https://docs.antismash.secondarymetabolites.org/glossary/#nrps),[transAT-PKS](https://docs.antismash.secondarymetabolites.org/glossary/#transat-pks),[betalactone](https://docs.antismash.secondarymetabolites.org/glossary/#betalactone) | 87619 | [fengycin](https://mibig.secondarymetabolites.org/go/BGC0001095/1) | 80% |
| Region 11 | [NRPS](https://docs.antismash.secondarymetabolites.org/glossary/#nrps) | 1364 | - | - |
| Region 12 | [NRPS](https://docs.antismash.secondarymetabolites.org/glossary/#nrps) | 1076 | - | - |
| Region 13 | [transAT-PKS](https://docs.antismash.secondarymetabolites.org/glossary/#transat-pks) | 34759 | [difficidin](https://mibig.secondarymetabolites.org/go/BGC0000176/1) | 40% |
| Region 14 | [NRPS](https://docs.antismash.secondarymetabolites.org/glossary/#nrps) | 21605 | [plipastatin](https://mibig.secondarymetabolites.org/go/BGC0000407/1) | 30% |
| Region 15 | [terpene](https://docs.antismash.secondarymetabolites.org/glossary/#terpene) | 21883 | - | - |
| Region 16 | [T3PKS](https://docs.antismash.secondarymetabolites.org/glossary/#t3pks) | 41100 | - | - |
| Region 17 | transAT-PKS-l | 463 | difficid | 53% |
| Region 18 | [terpene](https://docs.antismash.secondarymetabolites.org/glossary/#terpene) | 20740 | - | - |
| Region 19 | [PKS-like](https://docs.antismash.secondarymetabolites.org/glossary/#pks-like) | 41244 | [butirosin A / butirosin B](https://mibig.secondarymetabolites.org/go/BGC0000693/1) | 7% |
| Region 20 | [NRPS](https://docs.antismash.secondarymetabolites.org/glossary/#nrps) | 25552 | [surfactin](https://mibig.secondarymetabolites.org/go/BGC0000433/1) | 39% |
| Region21 | [NRPS](https://docs.antismash.secondarymetabolites.org/glossary/#nrps) | 28103 | [surfactin](https://mibig.secondarymetabolites.org/go/BGC0000433/1) | 47% |

**Tab. S4** Gene cluster of secondary metabolites of strainTC-10 predicted by antiSMASH

| Cluster | Type | Length (bp) | The Closest BGC | Similarity |
| --- | --- | --- | --- | --- |
| Region 1 | [betalactone](https://docs.antismash.secondarymetabolites.org/glossary/#betalactone) | 22671 | [mycosubtilin](https://mibig.secondarymetabolites.org/go/BGC0001103/1) | 20% |
| Region 2 | [ectoine](https://docs.antismash.secondarymetabolites.org/glossary/#ectoine) | 21229 | [ectoine](https://docs.antismash.secondarymetabolites.org/glossary/#ectoine) | 75% |
| Region 3 | [RRE-containing](https://docs.antismash.secondarymetabolites.org/glossary/#rre-containing) | 21229 | - | - |
| Region 4 | [siderophore](https://docs.antismash.secondarymetabolites.org/glossary/#siderophore) | 17664 | - | - |
| Region 5 | [T1PKS](https://docs.antismash.secondarymetabolites.org/glossary/#t1pks) | 47406 | - | - |
